# Sequence analysis of Discoknowium, an A5 mycobacteriophage

**DOI:** 10.1128/MRA.00920-23

**Published:** 2023-12-04

**Authors:** Kate B. R. Carline, Megan S. Fleeharty, Lin Fang, Mahima Shijo, Mitchell K. Doherty, Zoe A. Riddick, Marcus O. Royster, Amiyah R. Stukes, Bilalay V. Tchadi, Alana N. Thomas, Kurt E. Williamson, Margaret S. Saha

**Affiliations:** 1 Department of Biology, William & Mary, Williamsburg, Virginia, USA; Queens College, Queens, New York, USA

**Keywords:** Discoknowium, mycobacteriophage

## Abstract

Discoknowium is a temperate A5 bacteriophage that infects the bacterial host *Mycobacterium smegmatis*. Isolated from a rat fecal sample, Discoknowium’s genome is 50,222 bp in length, contains 84 genes and 1 tRNA, and shares 82%–98% nucleotide identity with other A5 subcluster phages.

## ANNOUNCEMENT

A growing body of research has documented the importance of bacteriophages in the mammalian microbiome, not only for understanding microbial dynamics but also for therapeutic applications (1 – 4). We therefore employed a rat fecal sample to isolate phages that infect *Mycobacterium smegmatis*, from a genus with pathogenic members that are increasingly developing antibiotic resistance (5 – 8).

**Fig 1:**
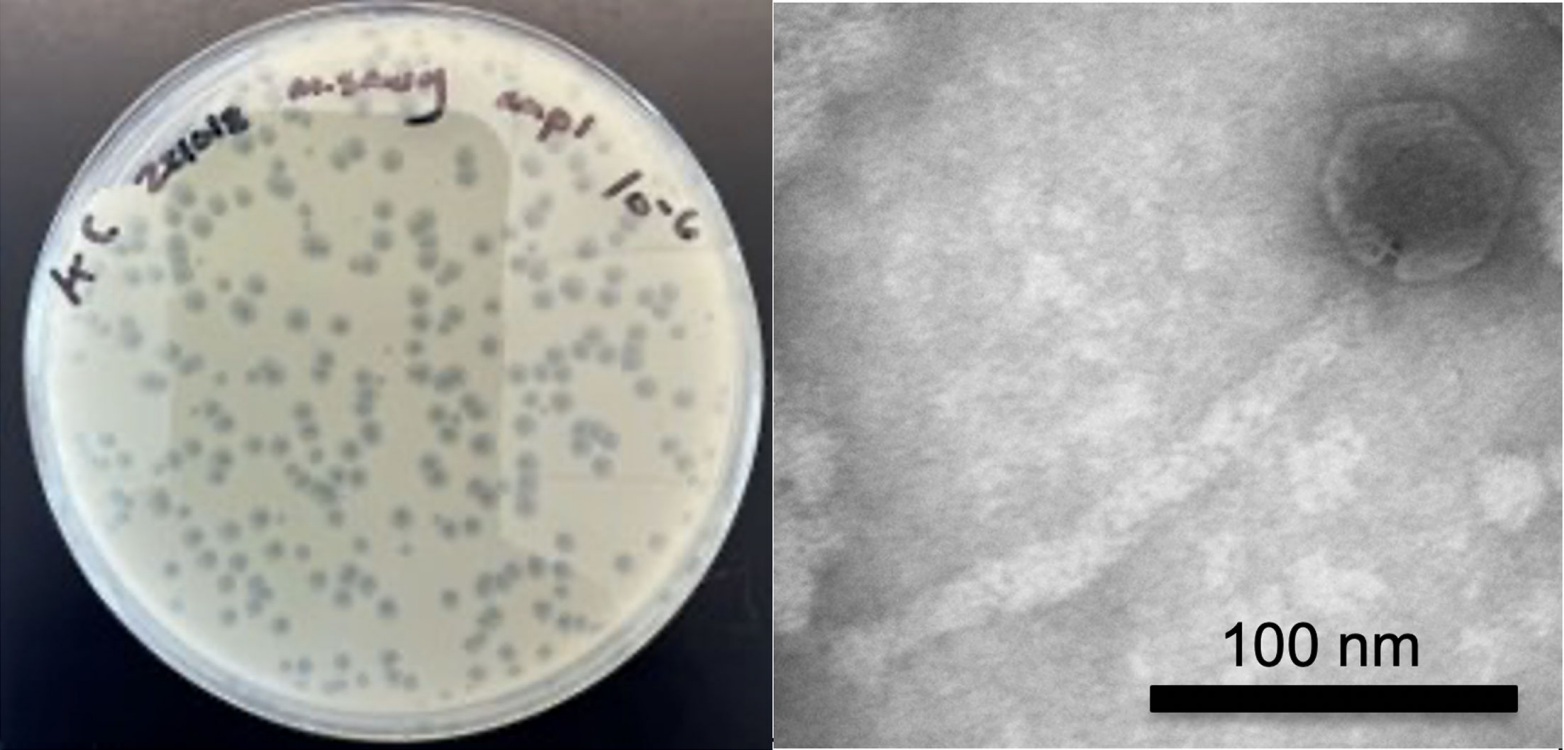
Plaque and transmission electron microscopy (TEM) images of Discoknowium. Left: Discoknowium forms clear plaques. Right: negative-stain (uranyl acetate, 1%) transmission electron microscopy performed using a Philips FEI CM10 revealed a siphovirus with an icosahedral head of ~70 nm (67–73 nm, *n* = 5) in diameter and a ~180 nm (174–185 nm, *n* = 5) flexible tail. Scale bar is 100 nm.

The mycobacteriophage Discoknowium was isolated using a standard enrichment protocol as detailed in the Phage Discovery Guide (9). Briefly, 1 g of a fecal sample from a Sprague Dawley rat obtained from the William & Mary vivarium in the Integrated Science Center on 4 September 2022 was incubated with 5 mL of *M. smegmatis* culture and 45 mL of 7H9 media for 48 h with shaking at 37°C. The culture was then filtered (0.22 µm), and the filtrate was plated in 7H9 top agar with *M. smegmatis*, resulting in clear plaques after 48 h at 37°C (Fig. 1). Discoknowium was purified through three rounds of plating. Negative-stain transmission electron microscopy reveals a siphovirus morphotype (Fig. 1).

Following DNA extraction using phenol-chloroform and ethanol precipitation (10), Discoknowium DNA was prepared for sequencing using the NEB Ultra II Library Kit and sequenced at the Pittsburgh Bacteriophage Institute using an Illumina MiSeq Sequencer (v3 reagents, single-end, and 150 base reads) to generate 80.4 million bases resulting in ~1,744 fold coverage. Newbler (v2.9) and Consed (v29) were used for assembly and finishing (11), yielding a genome of 50,222 base pairs with 60.9% GC content and a 3’ single-stranded ends (CGGGAGGTAA).

The genome was annotated using DNA Master (version 5.23.6) and PECAAN (version 20221109) following the Phage Genomics Guide (12). Translational start sites were verified using the coding potential predicted by GeneMark (13, 14), and the similarity of start sites in homologs was identified using Starterator (http://phages.wustl.edu/starterator/) and BLASTp (15). tRNAs were identified using Aragorn v1.2.41 (16) and tRNAscan (17). Putative gene functions were assigned based on predictions from HHPred (using the PDB_mmCIF70, NCBI_CD, SCOPe70, and pFAM-A as databases) and results from BLAST and Phamerator for highly similar genes (18, 19). Default settings were used for all software.

The Discoknowium genome encodes 1 tRNA and 84 protein-coding genes, 36 of which could be assigned functions that are involved in virion structure and assembly, DNA replication, lysis, and lysogeny. The first 29 genes and gene 31 are transcribed rightward, while the remainder are transcribed leftward. Based on gene content similarity (GCS) of at least 35% to phages in the Actinobacteriophage database (www.phagesDB.org), Discoknowium was assigned to subcluster A5, where it shares full GCS with Phlorence with the exception of one gene that it lacks (*Phlorence_58*). At the nucleotide level, Discoknowium shares 98% identity with Phlorence. Using NCBI BLAST (v2.14.0) and the BLOSUM62 matrix, we identified 298 nucleotide differences in the coding regions between these two phages, 114 of which result in synonymous differences, while 184 result in non-synonymous differences that present as either conservative amino acid differences (105) or non-conservative differences (79). Finally, we note that the intergenic region between genes 83 and 84 in Discoknowium is predicted by DNA Master to contain promoters.

## Data Availability

Discoknowium is available at GenBank with accession no. OR475282 and Sequence Read Archive (SRA) no. SRX21748144.
